# Electric field–guided random-access DNA data storage

**DOI:** 10.1126/sciadv.aee4328

**Published:** 2026-06-05

**Authors:** Doyeon Lim, Taeseok Kang, Wonjin Lee, Zeeshan Haider, Seunghwan Noh, Minsang Yu, Xiaohua Huang, Hyuk Soo Eun, Youngjun Song

**Affiliations:** ^1^ Department of Nano-Bioengineering, Incheon National University, Incheon 22012, Republic of Korea.; ^2^ Department of Intelligent Semiconductor Engineering, Incheon National University, 119 Academy-ro, Incheon 22012, Republic of Korea.; ^3^ Department of Bioengineering, Northeastern University Boston, MA 02115, USA.; ^4^ AptaBio Inc., 1307 Tower Bldg., Heungdeok IT Valley, Yongin 16954, Republic of Korea.; ^5^ Department of Bioengineering, University of California, San Diego, La Jolla, CA 92093, USA.; ^6^ Department of Internal Medicine, School of Medicine, Chungnam National University, Daejeon 35015, Republic of Korea.; ^7^ Department of Gastroenterology, Chungnam National University Hospital, Daejeon 35015, Republic of Korea.; ^8^ Standard Bioelectronics. Co., 511 Michuhol tower hall tower, Incheon 21999, Republic of Korea.

## Abstract

DNA offers the promise of high-density, long-term storage, yet current systems face limitations due to polymerase chain reaction–based manual workflows that are inherently slow, error prone, and difficult to scale for practical applications. Here, we present an electric field–guided DNA pool elongation system that addresses these challenges through molecular data control. Our electric field–driven DNA memory chip integrates immobilization for encoding with reusable synthesis access capabilities. Electric field–driven primer access and DNA synthesis were evaluated through position-specific primer hybridization combined with elongation at room temperature, maintaining high fidelity while dramatically reducing access times. This electric field–driven approach exhibited linear degradation, projected to exceed 10^5^ reuse cycles. Using this method with a common primer, we successfully stored and retrieved different DNA pools, enabling one-step next-generation sequencing library preparation. We further retrieved a 0.2–megabyte three-dimensional object encoded in 1339 unique strands, achieving 96.6% perfect matching. The system demonstrates chip-level capacities approaching 1.1 × 10^9^ molecules per electrode, representing a significant advancement toward practical, scalable DNA data storage.

## Teaser

Electric field–guided molecular control enables rapid, reusable DNA memory access and supports scalable data storage architecture.

## INTRODUCTION

DNA represents a promising next-generation data storage medium, providing unprecedented information density of up to 455 exabytes per gram, stability over millennial timescales, and intrinsic replication functionality compared to conventional silicon-based storage technologies (*1*–*3*). Since Church *et al.* (*4*) first demonstrated encoding a book in DNA, significant progress has been made in expanding storage capacity and functionality. Microsoft and the University of Washington researchers successfully stored over 200 megabytes of data and demonstrated random access capabilities (*5*). Recent studies have begun exploring the integration of DNA storage with computational capabilities, suggesting the potential for DNA-based computing architectures (*6*–*10*). DNA demonstrates significant potential to address the exponential growth in global data generation, which is projected to exceed 527.5 zettabytes by 2029 (*3*, *11*–*13*).

Despite these advances, current DNA data storage technologies face critical limitations that hinder practical implementation. First, polymerase chain reaction (PCR)–based access methods suffer from limited selectivity in data retrieval and introduce amplification bias and errors during the readout process (*14*–*16*). Second, existing DNA storage systems rely heavily on manual operations by skilled technicians for PCR preparation, primer design, and sequencing setup—factors that severely limits scalability and increases the likelihood of human error (*17*). For instance, typical next-generation sequencing (NGS) sample preparation requires 6 hours of labor-intensive procedures, creating significant bottlenecks in data access speed (*14*, *18*).

To address these PCR-related limitations, recent random access approaches have explored various encapsulation and particle-based strategies as alternatives to amplification-dependent retrieval. Boolean barcode sorting using silica capsules enables PCR-free selection but requires approximately 5 days for encapsulation and fluorescence-activated sorting (*19*). Thermoresponsive microcapsules allow repeated multiplexed access through temperature-controlled permeability but still depend on overnight DNA fixation and subsequent PCR amplification (*20*). Metal-organic frameworks achieve rapid 10-min encapsulation with flow cytometry–based selection yet remain limited to 84 reuse cycles (*21*). Photonic microspheres offer fluorescence-free indexing with high density (22.6 exabytes/g) but require approximately 72 hours for microsphere preparation followed by standard 6-hour NGS protocols (*22*). These particle-based approaches, while improving selectivity, fundamentally cannot eliminate the time-consuming NGS library preparation step that dominates the total data access latency, nor do they address the inherent lack of parallel processing capabilities in single-pool architectures (table S1).

Beyond these encapsulation strategies, electrode-based interfaces have also been explored as alternatives to enable electronic control over molecular operations (*23*–*25*). One approach uses digital microfluidic control to electrically manipulate and selectively retrieve stored DNA (*26*); however, this method is constrained by time-consuming resuspension procedures and inherent limitations in droplet routing pathways, making simultaneous access to multiple data pools difficult and restricting it to single-use operations. Other approaches have focused on direct DNA synthesis on electrode arrays, where individual sequences are synthesized per electrode (*27*, *28*), but these platforms are limited to writing single-stranded sequences encoding only individual data units per electrode, resulting in low data density and the same single-use restriction. While both chemical and enzymatic electrode synthesis methods have been developed, these remain fundamentally confined to writing operations and cannot address the data retrieval challenge (table S2). These architectural constraints and operational restrictions collectively prevent DNA storage from achieving the rapid, automated, and scalable access required for practical applications.

To overcome these fundamental challenges, a paradigm shift is needed that eliminates manual dependencies through electronic precision control while enabling parallel molecular operations. Here, we present an implementation of electric field–driven DNA elongation and immobilization that overcomes these restrictions, establishing a novel interface between molecular biology and electronics. Unlike conventional chemical or enzymatic methods, our system achieves position-specific DNA synthesis and immobilization through precise, reusable electric field control, demonstrating novel direct electrical manipulation of DNA data molecules. This approach fundamentally differs from existing electrode-based DNA synthesis methods—While prior work focused on writing new sequences through electrochemical deprotection, our system enables selective reading and retrieval from prestored DNA pools through electric field–guided hybridization. The result is a platform that achieves greater than10^5^ reuse cycles (compared to single-use in writing systems), reduces NGS preparation time from 6 hours to 50 min through direct adapter incorporation and enables common primer usage across different electrode positions—capabilities that are impossible with conventional encapsulation or synthesis-focused electrode approaches. This electric field–driven DNA synthesis chip enables spatially selective operations on electrode arrays, offering fully automated DNA data access and processing that transforms DNA storage into a practical, scalable technology platform.

## RESULTS

### Electric field–driven DNA synthesis onto microelectrode arrays

**Fig. 1.**
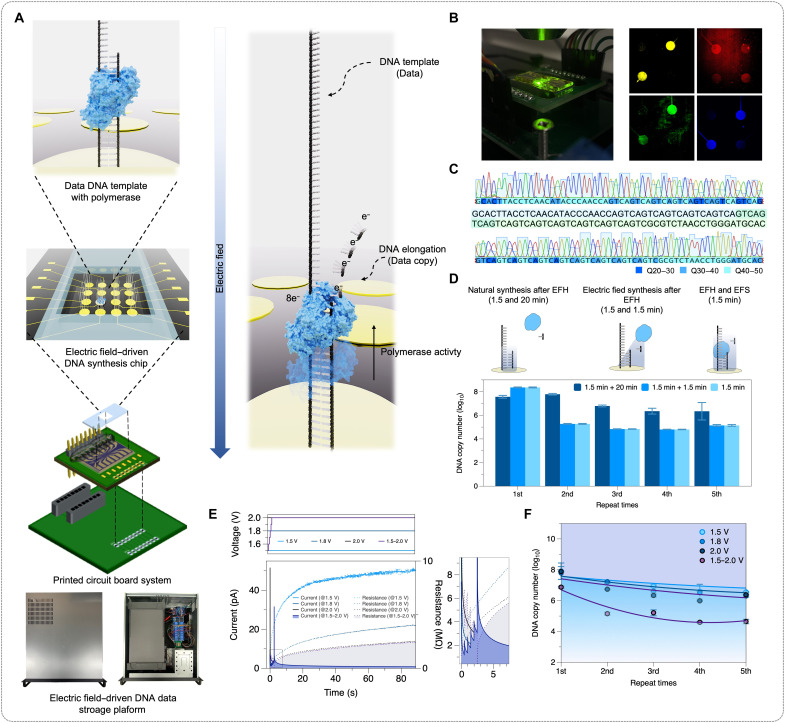
Electric field–driven DNA synthesis on a microelectrode array. (**A**) Schematic illustration and system image of the electrode-addressable DNA synthesis. (**B**) Sequential elongation by tracking fluorescent nucleotides (A, T, G, and C). (**C**) Sanger sequencing result. (**D**) qPCR analysis of DNA production efficiency across electric field-driven protocols (EFH, electric field–driven hybridization; EFS, electric field–driven synthesis). (**E**) Electrochemical response analysis under variable voltage. (**F**) qPCR analysis of electric field–driven synthesis efficiency across various electrical potentials with repeated synthesis trials (*n* = 5).

For DNA data storage, we introduce an electric field–driven DNA synthesis chip that enables selective access to DNA molecules (Fig. 1). Building on our previous studies (*29*, *30*), we established an electrochemically stable DNA-immobilized microelectrode surface (fig. S1) with optimized spatial configurations for electric field–guided polymerase activity to facilitate in situ DNA synthesis. Unlike conventional DNA synthesis methods such as PCR, our electric field–driven approach enables spatial DNA synthesis, addressing selective accessibility and electrochemical stability for the DNA polymerases. Notably, polymerase accessibility is further improved by introducing dedicated spaces during the DNA hybridization process, enabling seamless DNA synthesis directly on the active electrode surface. Figure 1A shows the microelectrode array platform and the electric field–driven DNA synthesis. First, primer DNA was selectively hybridized onto the immobilized target DNA on the electrode surface through electric field control for 1.5 min. Next, complementary DNA was synthesized directly on the electrode surface using a polymerase and deoxyribonucleoside triphosphates (dNTPs) for 20 min. We chose the Klenow fragment for its robust polymerase activity at room temperature, enabling the bypass of conventional heating steps. After synthesis, the DNA was extracted via a denaturation process and subsequently analyzed by sequencing. To the best of our knowledge, this electric field–guided DNA synthesis approach has not been previously reported. In particular, the Klenow fragment (with a net charge of 8 e^−^) can move in the direction opposite to a positive electric field because of its enzyme motor activity. Moreover, negatively charged dNTPs (with a net charge of 4 e^−^) are locally concentrated around the electrode, maintaining concentrations sufficient to support enzymatic DNA synthesis. Therefore, we performed comprehensive characterization under various experimental parameters to optimize the reaction conditions. To monitor selective spatial DNA synthesis in real time, we visualized sequential elongation by tracking fluorescently labeled nucleotides (A, T, G, and C) at each base incorporation step on the electrode surface (Fig. 1B). This real-time visualization clearly demonstrated stepwise nucleotide incorporation, indicating precise and efficient DNA synthesis under electric field conditions. The synthesized DNA was analyzed by Sanger sequencing, which confirmed high sequence fidelity to the original template (Fig. 1C). We further confirmed the DNA strand [100 base pairs (bp)] synthesis and denaturation from electrodes, using Cy3–deoxycytidine triphosphate (dCTP) incorporation (fig. S2).

**Fig. 2.**
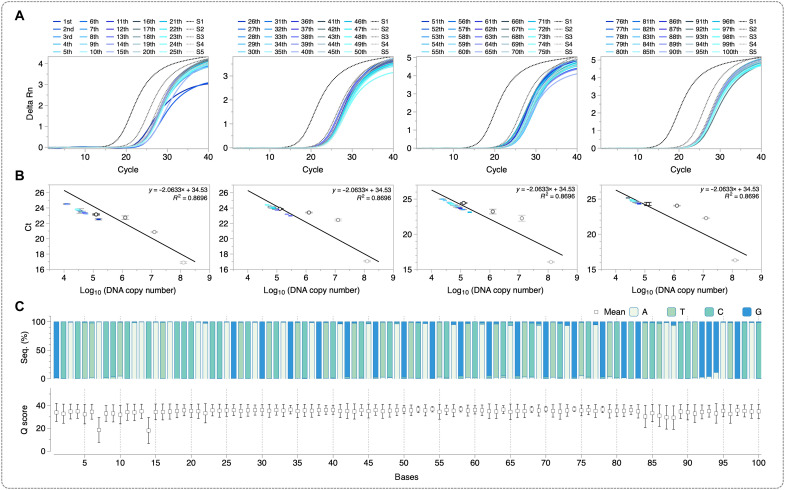
100-cycle repeated electric field–driven DNA synthesis. (**A**) qPCR analysis results of the 1st to 100th synthesized DNAs. (**B**) Standard curves for quantitative comparison. (**C**) Sequencing results for the 100th synthesized DNA. (top, consensus sequence; bottom, quality scores).

While the demonstrated process represents a novel approach for DNA synthesis, practical implementation for data storage applications requires further optimization. In particular, improvements are needed to achieve rapid access times and to ensure continuous molecular availability. To facilitate rapid DNA production, we examined the kinetics and catalytic efficiency of polymerases under electric field–compatible conditions. Figure 1D shows quantitative PCR (qPCR) results across three experimental conditions: a standard synthesis protocol involving 20 min of synthesis after 1.5 min of electric field–driven hybridization, an abbreviated protocol with 1.5 min of synthesis following electric field–driven hybridization, and a simultaneous protocol combining synthesis and electric field–driven hybridization for 1.5 min. Our preliminary findings show that simultaneous electric field–driven hybridization and synthesis substantially decrease the reaction time by 93%, albeit with a slight reduction in DNA production efficiency, which highlights potential benefits for rapid DNA production. Although this simplified synthesis protocol can be further optimized, we maintained a sufficient reaction duration in this study to support future applications such as longer DNA synthesis (*31*). In addition, we examined the impact of different electric field potentials on synthesis efficiency over repeated cycles (Fig. 1E). The optimal efficiency was observed at a potential of 1.5 V, yielding 3.47 × 10^6^ copies at the fifth cycle. Notably, synthesis efficiency significantly decreased at higher potentials, with 1.95 × 10^6^ and 0.95 × 10^6^ copies at 1.8 and 2.0 V, respectively (Fig. 1F). This decrease is likely due to electrochemical degradation of the electrode surface or compromised DNA stability. To minimize electrochemical degradation while maximizing DNA synthesis efficiency, we used a stepwise voltage-increment protocol, gradually raising the potential from 1.5 to 2.0 V in 0.1-V increments. Although this resulted in a slight reduction in DNA copy numbers (7.24 × 10^6^ at the first cycle), the approach effectively reduced sudden current spikes observed at higher voltages. To evaluate the long-term sustainability of molecular accessibility, we developed a degradation model based on quantitative analysis of 100 consecutive cycles of DNA production (Fig. 2, A and B), allowing us to monitor changes in electrochemical capacity over time. On the basis of a linear-regression degradation model (Eq. 1), we project that the system will retain functional integrity (≈1.95 × 10^6^ copies) for up to ~100,000 operational cycles

$${\text{log}}_{10}N_{n}={\text{log}}_{10}N_{0}-r\cdot n\;\left(r=3.1\times 10^{-5},R^{2}=0.997\right)$$ (1)

where $N_{n}$ is the remaining number of DNA copies after n cycles, $N_{0}$ is the initial number of DNA copies, r is slope of the decay, and n is the number of operational cycles.

To further validate the fidelity of our system, we performed NGS on the 100th DNA synthesis product (Fig. 2C), which confirmed high sequence accuracy and integrity. The NGS results obtained from the primer-based approach confirmed the feasibility of our method for selective DNA synthesis. These findings highlight the core significance of this platform—DNA strands can be selectively synthesized only on the desired microelectrode surfaces. This highlights the importance of implementing an independent immobilization process for each microelectrode to achieve spatially selective DNA data storage.

### Selective electric field–driven DNA memory access

**Fig. 3.**
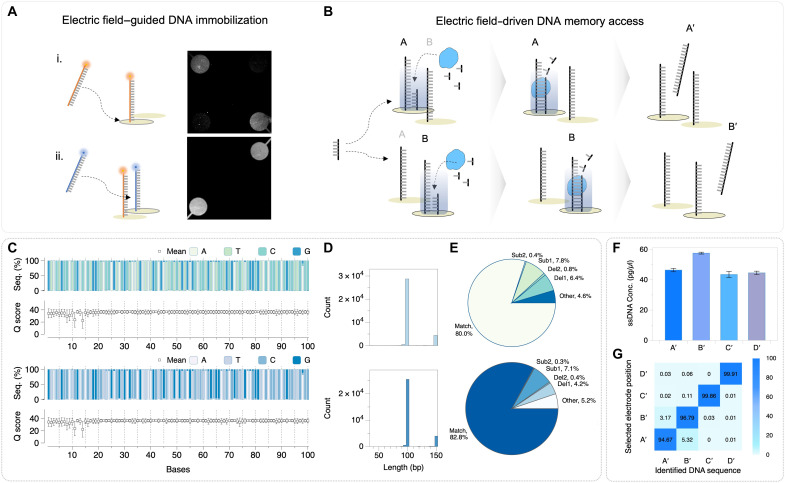
Selective electric field–guided DNA immobilization and electric field–driven DNA memory access. (**A**) Schematic illustration and fluorescence image of selective electric field–guided DNA immobilization. (**B**) Schematic illustration for electric field–driven DNA memory access. (**C**) Base sequence composition and quality score by position in NGS read. (**D**) Read length histograms from NGS analyses. (**E**) Pie chart showing the distribution of deletions and substitutions in selectively synthesized DNA. (**F**) Quantitative results of selectively synthesized DNAs. (**G**) NGS analysis of cross-talk in the four sequences.

For selective DNA synthesis on these electrodes, an independent immobilization process is required at each microelectrode position. However, conventional methods for immobilizing chemically modified DNA onto electrode surfaces typically require prolonged reaction times. Moreover, positioning molecules from solution at each microelectrode is extremely challenging to achieve in practice. To address this, we developed an electric field–guided DNA immobilization method, achieving rapid attachment of amine-DNA to amine-tethered electrodes within 20 min. This approach offers significant temporal and spatial advantages compared to conventional thiol-based immobilization strategies (*29*, *32*). To evaluate this novel electric field–guided DNA immobilization approach, we demonstrated selective binding of two distinct amine-modified DNA sequences labeled with fluorescent dyes—FAM and Cy5. Figure 3A presents a fluorescent microscopy image of these sequentially immobilized DNA onto electrodes. The results clearly confirm successful spatially controlled attachment of the differentially labeled DNA sequences on a single chip, highlighting capability of the electric field–guided DNA immobilization method for precise access at defined microelectrode positions. We demonstrated the synthesis of two distinct DNA sequences using an electric field–driven method with the same primer (Fig. 3B). The clear, distinct sequences of these distinguishable DNA strands were confirmed by NGS results (Fig. 3, C to E). To further validate the selectivity of our approach, we extended this experiment to four distinct DNA sequences (fig. S3). The extracted DNA quantities from each electrode were approximately 40 to 60 ng/μl, and all four sequences were successfully amplified via PCR (Fig. 3F), confirming the functional integrity of the selectively immobilized templates. NGS analysis of these four-sequence experiments revealed that cross-electrode contamination remained below approximately 5.32% (Fig. 3G), confirming negligible crossover between spatially separated DNA molecules. Specifically, the NGS analysis of strand B′ contained only 3.17% of A′ (with C′ and D′ at 0.03 and 0.01%, respectively), while the highest crossover observed for strand A′ was from strand B′ at 5.32%. With this selective DNA immobilization methodology, we enable precise electric field–driven DNA memory access on our microelectrode array platform. This capability allows us to encode digital information directly into DNA sequences.

### DNA processing in memory

**Fig. 4.**
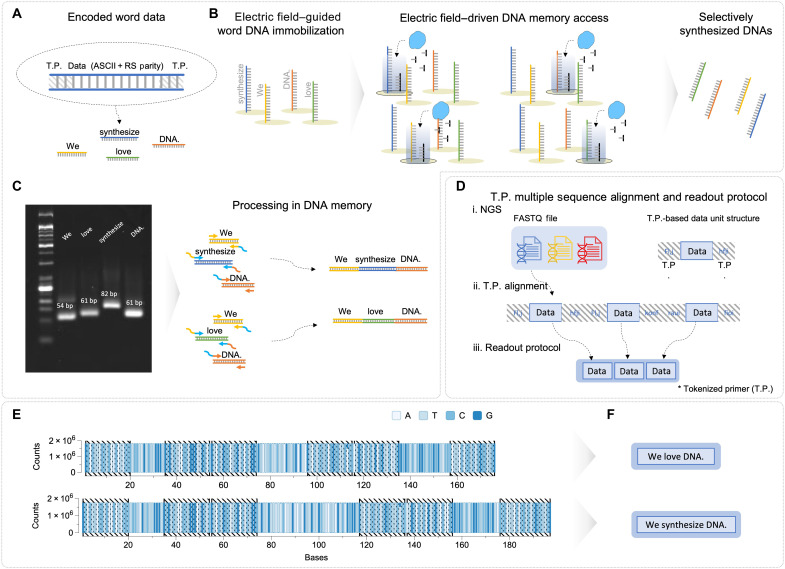
DNA processing in memory. (**A** to **C**) DNA data process using electric field–driven synthesis and assembly PCR. (**D**) Readout protocol of electric field access DNA memory. (**E**) NGS consensus results for assembled DNA data storage. (**F**) Readout data for DNA processing in memory.

Individual DNA data molecules were produced selectively on-chip following our word-based encoding scheme (fig. S4). In this process, input word data are first converted to American Standard Code for Information Interchange code (ASCII code) and then transformed into eight-bit binary format. To ensure data integrity, Reed-Solomon (RS) parity bits are generated using RS code (8,3) encoding rules, adding seven bits of error-correcting code redundancy bits per data block. The binary data are subsequently translated into DNA bases, and finally, tokenized primers (T.P.) are added to enable addressable retrieval. This encoding process yielded DNA sequences ranging from 54 to 82 nucleotides depending on the input word length (Fig. 4A). The resulting DNA data molecules serve not only as storage units but also as computational substrates that can be efficiently manipulated for DNA information processing through processing-in-memory capabilities via assembly PCR. The processing mechanism operates in two stages: First, individual DNA data molecules are selectively synthesized at designated electrodes through electric field–driven control (Fig. 4B); second, these molecules are released, collected, and concatenated via assembly PCR, where overlapping tokenized primer sequences serve as linkage points between fragments (Fig. 4C). For data readout, the processed DNA information undergoes token identification via primer-based sequence alignment scoring (Fig. 4D). This primer-based approach not only yields accurate DNA data retrieval but also enables effective data processing through the deployment of sequence pipelines using address identifiers. Through analysis of high-fidelity NGS results, we confirmed that our system successfully assembled word-level DNA memory molecules into various large-scale sentence-level constructs (Fig. 4, E and F). For instance, we can create “We love DNA.” or “We synthesize DNA.” by selectively synthesizing different DNA payloads at distinct electrode addresses, using the same primer. The electrode-controlled circuitry enables selective production of specific DNA data molecules from each address, while the subsequent assembly PCR concatenates these molecules on the basis of their designed primer address complementarity, creating new semantic combinations. The assembled DNA constructs achieved sequencing-compatible lengths of 176 nucleotides (We love DNA.) and 197 nucleotides (We synthesize DNA.), enabling ordered sequential word combinations through our electrode-based selective synthesis system. This ability to access and process data at various physical addresses using a single primer represents a core functionality of our electric field–based processing-in-memory system. This DNA processing-in-memory system demonstrated editing functionality at the word or token level, enabling the implementation of large-scale models, significantly outperforming single-bit manipulation methods (*33*).

This demonstrates not only data storage but also the potential for complex information processing within DNA memory systems. Our study presents three critical advancements in DNA memory technology. First, the electric field–guided DNA immobilization method enables independent DNA encoding at each microelectrode position, achieving rapid and spatially controlled attachment in just 20 min. This breakthrough significantly reduces processing time compared to conventional thiol-based methods while maintaining precise positional control. Second, the integration of DNA memory with semiconductor chip fabrication opens new avenues for postsynthesis gene editing and molecular data manipulation at the chip level. This convergence of biotechnology and semiconductor engineering establishes a scalable platform for complex DNA-based computation. Third, the implementation of selective binding using identical primers across different electrode addresses enables improved DNA synthesis control. This capability to synthesize desired genes through primer-based selection offers significant advantages for NGS preparation workflows, where uniform primer usage can considerably simplify library construction while maintaining sequence diversity. Together, these advances position our electric field-driven DNA memory system as a transformative technology for next-generation data storage and processing applications.

### Selectively access for parallel DNA memory

**Fig. 5.**
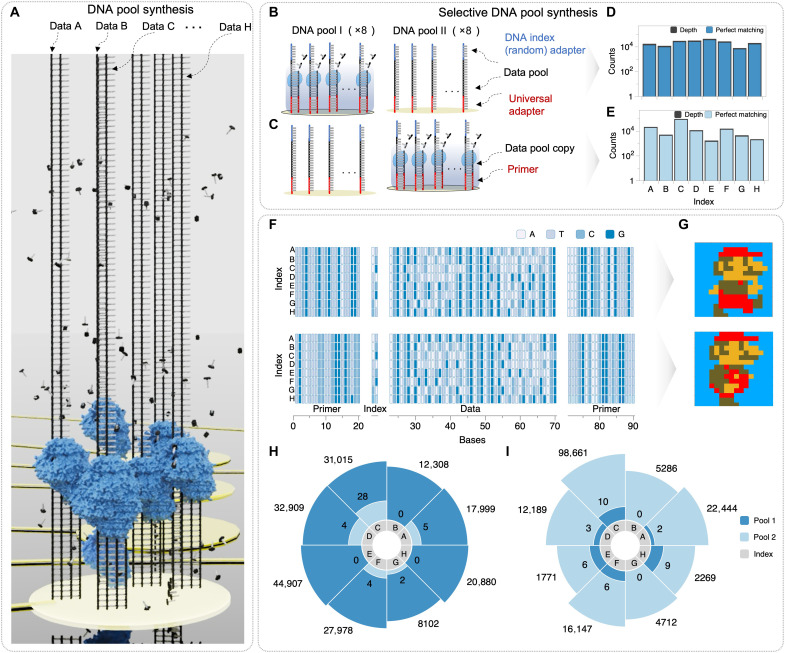
Selectively electric field–guided DNA pool data access with NGS adapter. (**A**) Schematic illustration for randomly immobilized DNA pool synthesis. (**B** and **C**) Schematic illustration of DNA pool I (B) and DNA pool II (C). (**D** and **E**) NGS quantification per index sequences for selective DNA pool synthesis of DNA pool I (D) and DNA pool II (E). (**F** and **G**) NGS consensus results (F) and decoded pixel-art images for selectively electric field–accessed DNA pool data with NGS adapter (G). (**H** and **I**) NGS analysis for cross-talk between different DNA pools. (The values are presented on a logarithmic scale.

Despite the advantages in the synthesis-based data encoding of single-electrode approaches for base-level (two bits) synthesis (*27*, *28*), they present fundamental limitations to storage and access large-volume DNA data pools. Moreover, although NGS techniques are essential for accurately reading DNA data pools, sequencing requires at least 6 hours for sample preparation and is prone to manual errors. To address these issues, we leveraged electric field–driven DNA memory access to facilitate both selective access and parallel DNA memory operations, significantly enhancing efficiency and scalability in DNA-based data storage and retrieval (Fig. 5A).

In the initial experiment, we verified selective access by adapter-mediated immobilization of DNA under an electric field. Through the selective immobilization of DNA data pools containing sequencing adapters, we successfully recovered the DNA data pools demonstrated in our earlier work (*34**,* *35*), as shown in Fig. 5B. NGS analysis revealed that the parallel 213-nucleotide DNA data pools (×8) were uniformly recovered with high fidelity (gel electrophoresis verification is provided in fig. S5), enabling accurate reconstruction of the original pixel-art image using only consensus sequencing without additional error correction methods (fig. S6 for detailed decoding method). To quantify the spatial selectivity of our identical primer-based approach, we performed NGS analysis of cross-electrode sequence distribution (Fig. 3G), confirming negligible crossover between electrodes despite using common primers. Furthermore, our experimental approach enables direct sequencing after quantitative control through PCR, allowing us to complete the sample preparation process within 50 min. This demonstrates the enhanced efficiency of our electric field–driven approach (fig. S7 for detailed protocol and table S1).

Having established the fundamental capability of selective DNA pool recovery, we next investigated the spatial selectivity of our platform to enable fully independent parallel operations. This second experiment aims to demonstrate parallel DNA memory operations through identical primer-based spatial selectivity. To evaluate the independence of primer sequences across electrodes, we demonstrated electric field–driven DNA synthesis using identical primer sequences on separate electrodes with individually immobilized different DNA templates (Fig. 5, B and C). The NGS results revealed remarkable sequence fidelity with 99.999% accuracy matching to their respective original templates, validating the effectiveness of our selective approach. These results demonstrate that despite sharing identical primer sequences in the same reaction solution, the electric field enabled selective hybridization exclusively at specifically activated electrodes. The spatial selectivity established by our approach represents a critical advancement for facile access to DNA data storage, enabling accurate and selective data retrieval through targeted DNA synthesis. Specifically, our methodology significantly simplifies sequencing workflows by using common primer sequences across multiple electrodes, which is especially beneficial for situations that require uniform primer-based data selection. With the spatial selectivity validated, we proceeded to demonstrate the full potential of our system through integrated parallel DNA memory operations and complete data retrieval. Last, we demonstrated the complete data retrieval pipeline through NGS-based decoding of the spatially selected DNA pools. The parallel DNA data pools, which were selectively extracted from the electrodes, were processed according to simple NGS protocols, with distinct NGS indices assigned to each pool for simultaneous multipool sequencing. The NGS analysis revealed that both spatially selected DNA pools maintained uniform and high-fidelity sequence reads (Fig. 5, D and E). To validate the semiconductor chip–based molecular operations, we used two distinct DNA pools encoding different Mario pixel-art images, using index-based barcoding for sequencing sample separation and primer-based sequences for actual data identification (Fig. 5F). This index-based approach enabled electrode-specific data retrieval, where each electrode’s DNA pool was independently accessed and decoded without interference from adjacent electrodes. This classification scheme enabled precise data analysis and systematic management. The NGS reads resulted in the perfect reconstruction of the original pixel-art images from their respective DNA pools, confirming the complete functionality of our integrated system (Fig. 5G). The successful parallel decoding represents the final critical component in our DNA data storage architecture in which semiconductor chip–based molecular operations are seamlessly interfaced with digital data recovery, thereby establishing a complete pathway from on-chip storage to DNA data retrieval. Quantitative analysis further confirmed the spatial selectivity of our system. The DNA molecules remain spatially confined to their designated electrodes during electric field–driven operations (Fig. 5, H and I) and the cross-electrode interference is negligible with a crossover rate of only 0.4%.

Our semiconductor chip–based molecular operations achieve remarkable advances in spatial selectivity, and parallel pool data scalability by means of electric field–driven DNA synthesis. Our chip architecture achieves a theoretical parallelism of 1.1 × 10^9^ DNA molecules per electrode, with experimental verification of 1339 sequences per electrode. Conventional electrochemical deprotection approaches (*27*, *28*) and electrowetting-based approaches (*25*) are each limited to a single sequence per electrode. Resuspension-based digital microfluidic approaches (*26*) rely on large electrode dimensions (2 mm by 2.7 mm) coordinated adjacent electrode actuation. These conventional approaches have a significantly lower storage density (3.703 × 10^−6^ KB/μm^2^) compared to our system (6 × 10^−3^ KB/μm^2^) (table S2). Furthermore, our 16-plex parallel operation with reusability represents a proof-of-concept demonstration that is readily extensible to higher-throughput configurations by increasing the number of independently addressable electrodes in the arrays (table S1). This selective parallel access to DNA data molecules using adapter primers provides two critical advantages: the rapid and systematic retrieval of stored information while eliminating dependence on error-prone and time-consuming manual processing steps. The adapter primer technology serves not merely as an experimental approach but as a practical retrieval mechanism that transforms DNA data storage accessibility. This advance directly addresses the previously identified challenges of lengthy NGS preparation processes and manual handling errors. The elimination of manual process dependencies leads to enhanced system reliability, establishing a foundation for automated DNA data operations. By integrating electric field–driven selection with adapter-based identification, our approach achieves automated precision in accessing specific DNA data pools from complex mixtures, representing a significant advancement toward practical DNA data storage systems.

### High-scale DNA memory pools

**Fig. 6.**
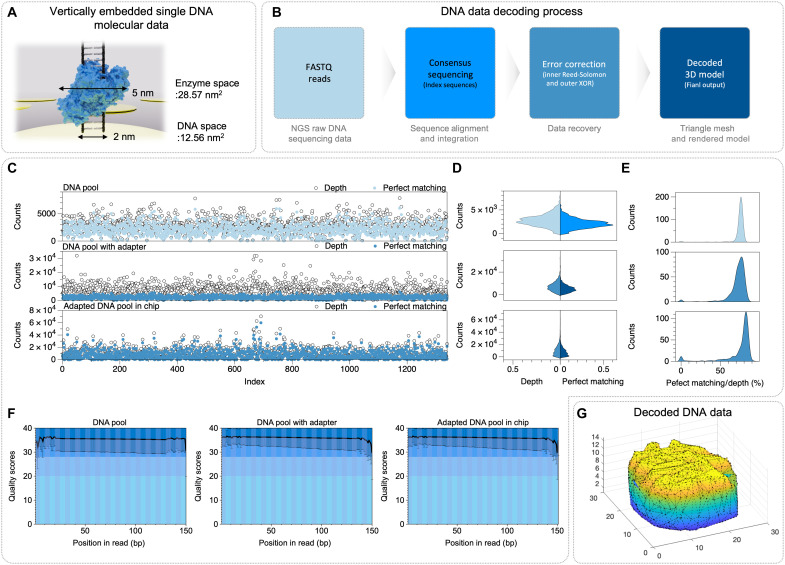
High-scale DNA memory pool and analysis of adapter sequence. (**A**) Schematic illustration for vertically embedded single DNA molecular data. (**B**) DNA data decoding process. (**C** to **F**) Comparative NGS analysis results for the original DNA data pool, adapted DNA data pool in solution, and adapted DNA pool in chip, showing sequencing depth and perfect match counts by index (C), distribution of their read counts (D), ratio of perfect matching to depth (E), and quality score along the read position (F). (**G**) Decoded 3D file image for adapted DNA pool in chip.

In our system, the payload capacity of randomly distributed DNA data molecules immobilized on the electrode surface was calculated as 0.035 copies/nm^2^, based on a spatial allocation of approximately 28.57 nm^2^ per molecule (Fig. 6A). This density theoretically enables storage of up to 1.1 × 10^9^ copies per single electrode. To evaluate the practical feasibility of electrode-based DNA data storage, we accessed and analyzed 1339 unique DNA data molecules encoding a 0.2-megabytes 3D cultural heritage file (fig. S8) using NGS methodologies, with our rapid presequencing workflow (50-min protocol for NGS library quantification).

To encode 3D spatial data into DNA sequences, positional coordinates and their connectivity were linearly arranged and translated into nucleotide sequences without compression. To enhance data integrity and prevent loss during processing, an XOR-based (XOR: Exclusive OR) redundancy scheme was applied to these sequences, improving their stability. For practical synthesis of these long DNA sequences in a pool-based system, the data were divided into fixed-length fragments. Each fragment was encoded as a 150-nucleotide sequence which include an index, RS error correction codes, and flanking primer sequences for manipulation. The encoding requires a total of 1339 unique DNA strands (Fig. 6B). The ligated 275 nucleotide DNA data pool with NGS adapter sequences (5′ amine) was electrically coupled to the selective electrode surface (gel electrophoresis verification is provided in fig. S5). For evaluating the molecular information fidelity, we conducted multilevel comparative analysis using NGS between the encoded DNA pool, the ligated DNA pool, and the DNA products synthesized and extracted from the electrically interfaced DNA pool on the selective electrode. Among the sequences analyzed via NGS (Fig. 6, C to F, and fig. S9), the chip based–DNA data storage with electrically interfaced DNA pool exhibited 96.56% perfect matching per index, with a median depth of 10,337.9 (bottom of Fig. 6, C and D), enabling content retrieval reliability. The encoded DNA pool, and the ligated DNA pool exhibited matching sequences coverage rate of 99.18 and 98.88%, while perfect matching rates of each index have similar values (74.29, 72.02, and 73.62%). The synthesis quality of electrode-immobilized DNA molecules was comparable slightly superior to that achieved by conventional methods (Fig. 6F). The high-quality anchored DNA on the chip enables precise downstream processes (fig. S9A). Through precise comparative analysis of sequence variations, we validated the reliability of the data molecule-based storage system and confirmed the integrity of the information throughout the electrical coupling process. The experimental results revealed near-uniform depth for each sequence, with very low rates of undersequenced or undetected sequences (fig. S9). This demonstrates that most stored content fragments were successfully recovered through sequencing, indicating low DNA data loss rates. Nevertheless, the increasing number of DNA pool losses due to the 2D electrode architecture in chip-based DNA pool access via electrical interface could be addressed through electrode structures using diverse geometries and materials. The 3D data were rapidly decoded by generating consensus sequences per index, while missing molecular data sequences were effectively reconstructed using XOR-based decoding methods (Fig. 6G).

## DISCUSSION

Because of the exponential growth of global data generation, DNA storage has emerged as a promising solution that demands revolutionary approaches beyond conventional silicon-based systems. However, many challenges remain. We have developed an electric field–driven DNA synthesis and memory access system that fundamentally transforms DNA data storage from a promising concept into a practical technology. Our approach pioneers the convergence of molecular biology and electronics. Precise electric field control enables spatially selective DNA synthesis and immobilization on microelectrode arrays. Through comprehensive experimental validation, we demonstrated electric field–guided DNA synthesis with high sequence fidelity with a 93% reduction in reaction time while maintaining synthesis efficiency. In this work, we confirmed synthesis of strand lengths of 213 nucleotides and 275 nucleotides that include NGS adaptor sequences for direct sequencing workflow compatibility.

Our approach encompasses key innovations that collectively transform DNA storage into a practical technology platform. First, we demonstrated electric field–driven DNA synthesis directly onto microelectrode arrays for spatially selective DNA synthesis. Unlike PCR-based random access DNA storage methods, our system allows targeted selection of DNA data from specific microelectrode locations while using identical common primers across all electrodes. Moreover, our platform provides direct, reusable electrical control over molecular operations without the limitations of single-use systems and concerns for molecular data loss. The system exhibited remarkable durability, projecting functional integrity for approximately 100,000 operational cycles based on our linear degradation model. More practically, we demonstrate that the selectively synthesized word-level DNA data can be manipulated through DNA processing-in-memory operations. Next, our addressable electrode-based DNA data storage system uses uniform primers across different electrodes. This dramatically improves workflow efficiency for NGS library preparation. The system eliminates manual dependencies through fully automated DNA access, addressing the 6-hour bottleneck of traditional NGS sample preparation. Last, the electric field–guided DNA immobilization enables encoding of DNA data pools at independent microelectrodes, dramatically improving data storage capacity. We demonstrated this capability through successful storage and retrieval of 0.2-megabyte data encoding 1339 unique DNA sequences, with 96.56% perfect matching per index and negligible cross-electrode interference. This proof-of-concept validates the system’s scalability potential for larger-scale implementations.

The successful integration of electronic control with molecular operations establishes a new paradigm for information storage and processing, bridging digital and biological systems. This work represents a fundamental shift in DNA data storage approaches, opening pathways toward automated electronic-molecular architectures that transcend manual DNA storage limitations.

## MATERIALS AND METHODS

### Chip preparation and the system

The microelectrode array chip was fabricated by a normal semiconductor fabrication process. The electrode array pattern was created on a SiO_2_ silicon wafer of 500-nm thickness (Silicon Technology Co., Kyowa, Japan) with a DNR-L300-D1 negative photoresistor (Dongjin Semichem Co., Seoul, Korea) by MDA–400LJ mask aligner (MIDAS System Co., Daejeon, Korea) with a custom-designed photomask (Microt Co., Seongnam, Korea). A 5-/100-nm thickness of Ti/Au was deposited by E-beam evaporation (UEE, ULTEC Co., Daegu, Korea). For the selective DNA immobilization, the gold pattern surface was treated with 10 mM cysteamine (Sigma-Aldrich, St. Louis, MO, USA) in buffer 99.9% ethyl alcohol (Duksan Pure Chemical Co., Ansan, Korea) for 3 hours at room temperature. Respectively, the gold pattern surface was treated with 100 mM BS^3^ (Thermo Fisher Scientific, Waltham, MA, USA) in DI (de-ionized) water for 20 min at room temperature. The 3′-tethered amine DNAs were ordered from Integrated DNA Technologies (IDT Inc., Coralville, IA, USA). The DNAs were immobilized onto the amine-activated microelectrode array chip, which was connected by a customized switch board to a Keithley 2450 source meter (Keithley Instruments Inc., Cleveland, OH, USA). For electric field–driven DNA immobilization and hybridization, the suspension conditions were prepared with 12.5 mM histidine (Sigma-Aldrich Co., St. Louis, MO, USA), 0.5× phosphate-buffered saline (Samchun Pure Chemical Co., Seoul, Korea), and 100 mM NaCl (Samchun Pure Chemical Co., Seoul, Korea) in DI water. The chip was wire bonded to a customized PCB (HK WEIKU Technology Company Limited, China) and epoxy-sealed by Loctite Co. (Rocky Hill, Conn, USA). The chip was controlled by the Arduino Megaboard 2560 R3 (Arduino Co., Somerville, MA, USA) with a 16-channel relay module board (Uctronics, Jiangsu, China). The custom software for the chip operating interface was developed by Visual Studio C++ (Microsoft Inc., Seattle, NW, USA) with the serial communication protocol. To monitor current and apply voltage to the electro-thermodynamic chip, the Keithley 2450 source meter (Keithley Instruments Inc., Solon, OH, USA) was used as the voltage source for the chip. The system was mounted in an Amaquest V3-500 server rack (Amaquest Inc., Seoul, Korea). The element morphologies of the samples were investigated using various imaging techniques. X-ray photoelectron spectroscopy (XPS) mapping images were acquired using a Nexsa-G2 XPS system (Thermo Fisher Scientific, Waltham, MA, USA), and EPMA images were acquired using a JXA-8530F EPMA (JEOL Ltd., Tokyo, Japan). Fluorescent images were captured using a DMRBE upright Leica microscope (Leica Microsystems, Wetzlar, Germany) equipped with a Prime 95B camera (Teledyne Photometrics, AZ, USA).

### Electric field–guided DNA synthesis and assembly PCR

All DNAs were ordered from Integrated DNA Technologies (IDT Inc., Coralville, IA, USA). The electric field–guided DNA synthesis was performed with the dNTP solution mix (New England Biolabs Inc., Ipswich, MA, USA) and Klenow fragment (New England Biolabs Inc., Ipswich, MA, USA). The synthesized DNA was washed with 50 mM NaCl buffer and then denatured in 10 μl 0.125 M NaOH or 95°C DI water. For the rich PCR product, template DNAs, which were synthesized by the electric field–guided DNA synthesis process, were amplified with 20 cycles in the dNTP solution mix (Thermo Fisher Scientific Inc., Waltham, MA, USA) and Taq polymerase (Thermo Fisher Scientific Inc., Waltham, MA, USA) by SimpliAmp Thermal Cycler (Thermo Fisher Scientific Inc., Waltham, MA, USA). In addition, the DNAs were assembled by a one-step assembly PCR process. For gel electrophoresis results, the 2.0% agarose gel (Sigma-Aldrich Co., St. Louis, MO, USA) in 1× TBE buffer ran for 40 min. The gels were scanned by an Amersham Typhoon 5 gel scanner (Cytiva Inc., Marlborough, MA, USA).

### Measurement of DNA

To monitor electric field–guided DNA synthesis on the chip, we used Mant–deoxyadenosine triphosphate, FAM–deoxyuridine triphosphate, Cy5-dCTP, ATTO 565–deoxyguanosine triphosphate obtained from Jena Bioscience (Jena, Germany). The synthesized DNA samples with fluorescence on the chip were observed using a DM RBE upright fluorescent microscope (Leica Microsystems, Wetzlar, Germany) equipped with a Prime 95B scientific complementary metal-oxide semiconductor image sensor (Teledyne Photometrics, AZ, USA). The quantities of the produced DNAs, repeated 100 times, were analyzed using a three-step approach with the PowerUP SYBR Green Master Mix (Thermo Fisher Scientific Inc., MA, USA) on the QuantStudio 5 Real-Time PCR system (Thermo Fisher Scientific Inc., Waltham, MA, USA). For Sanger sequencing, the DNA A sequence was analyzed using the 3730XL DNA Analyzer (Thermo Fisher Scientific Inc., Waltham, MA, USA) provided by Bionic. NGS was performed on 100-bp DNAs using the TrueSeq Nano library prep kit (Illumina Inc., San Diego, CA, USA) and the iSeq system (Illumina Inc., San Diego, CA, USA). DNAs ranging from 176 to 275 bp were analyzed using the MiSeq system (Illumina Inc., San Diego, CA, USA).

### Encoding and decoding of DNA sequences

The word DNAs were encoded by seven bits of ASCII with seven bits of correction RS code. The finalized sequences were defined with independent primer sequences. For decoding protocol, the NGS results, which have paired end sequencing were converted and sorted by primer sequences and analyzed by consensus sequencing. Two pixel-art images were encoded in eight 90-bp DNA pools, with a 2-bp index and two 20-bp primer sequences, using a previously customized codon-based code29. 3D cultural heritage datasets were encoded using a highly efficient 3D structural numerical method with inner RS codes and outer XOR redundancy sequences. (A comprehensive description of the encoding and decoding methods is provided Fig. 5B and fig. S8.). For the decoding process the NGS read sequences were clustered on the basis of primer set information. Excluding primer sequence information (paired-end sequencing), the read sequences were converted into digital information and decoded using RS codes. The decoded digital sequence reads were categorized by index sequences. Last, the 3D data information was recovered using the XOR redundancy sequences. The unrecovered points were adjusted using a modified interquartile range (IQR) method, where Q1 (the first quartile) represents the 25th percentile, and Q3 (the third quartile) represents the 75th percentile of the data. The lower bound was calculated as Q1 − 0.55 × IQR, and the upper bound as Q3 + 0.55 × IQR. The scripts for the encoding and decoding protocol were developed using MATLAB 2024a (MathWorks Co., Natick, MA, USA). All raw sequencing data and scripts used in this study were provided at GitHub (https://github.com/bioelectronicsbiooptics/Electric-field-guided-DNA-data-storage-chip).

### DNA sequencing

DNA sequences were obtained from Integrated DNA Technologies (IDT Inc., Coralville, IA, USA). The encoded DNA pools were ordered SurePrint oligonucleotide pools (Agilent Technologies Inc., Santa Clara, CA, USA). For ligation, the 5′ end of DNA sequences and encoded DNA pools were phosphorylated with T4 polynucleotide kinase (New England Biolabs Inc., Ipswich, MA, USA). The phosphorylated DNA sequences and encoded DNA pools were hybridized with splint DNA in the annealing process, in which the temperature was decreased from 95° to 30°C by 1°C min^−1^ in a SimpliAmp thermal cycler (Thermo Fisher Scientific Inc., Waltham, MA, USA). All DNA sequences and encoded DNA pools were prepared to achieve a final concentration of 50 fmol. The amplified DNA pools were then purified using Illumina sample purification beads (Illumina Inc., San Diego, CA, USA). The ligated with amine tethered DNA were processed by electric field–driven DNA synthesis. The denaturation sample were amplified with Master Mix (Roche, Basel, Switzerland). The enriched DNA library was purified using PCR purification kit (Qiagen Inc., Hilden, Germany) and finally quantified with Qubit dsDNA HS assay kit (Thermo Fisher Scientific Inc., Waltham, MA, USA), using Qubit 4 Fluorometer (Thermo Fisher Scientific Inc., Waltham, MA, USA). The final sample was then prepared for sequencing by following the iSeq100 System Guide. The final 50 pM samples were loaded in our Illumina iSeq100 (Illumina Inc., San Diego, CA, USA) with a 5% PhiX for a control spike-in. The DNA libraries were analyzed using the 150-bp paired-end protocol. Some DNA libraries were sequenced using the Illumina NovaSeq6000 system (Illumina Inc., San Diego, CA, USA) outsourced from Macrogen. (Macrogen Inc., Seoul, Korea), following the 150-bp paired-end protocol.

## Supplementary Materials

### This PDF file includes:

Figs. S1 to S9 Tables S1 and S2 References
