# Supplementary material for: Electric field–guided random-access DNA data storage
Source: Sci Adv. 2026 Jun 5;12(23):eaee4328. doi: 10.1126/sciadv.aee4328 (PMC13240191; doi:10.1126/sciadv.aee4328)
Supplement: Supplementary file 1 — Figs. S1 to S9 Tables S1 and S2 References [file sciadv.aee4328_sm.pdf]

Science Advances
AAAS

Supplementary Materials for

Electric field–guided random-access DNA data storage

Doyeon Lim *et al.*

Corresponding author: Xiaohua Huang, x2huang@ucsd.edu; Hyuk Soo Eun, hyuksoo@cnuh.co.kr; Youngjun Song, yjunsong@inu.ac.kr

Sci. Adv. **12**, eaee4328 (2026)
DOI: 10.1126/sciadv.aee4328

This PDF file includes:

Figs. S1 to S9
Tables S1 and S2
References

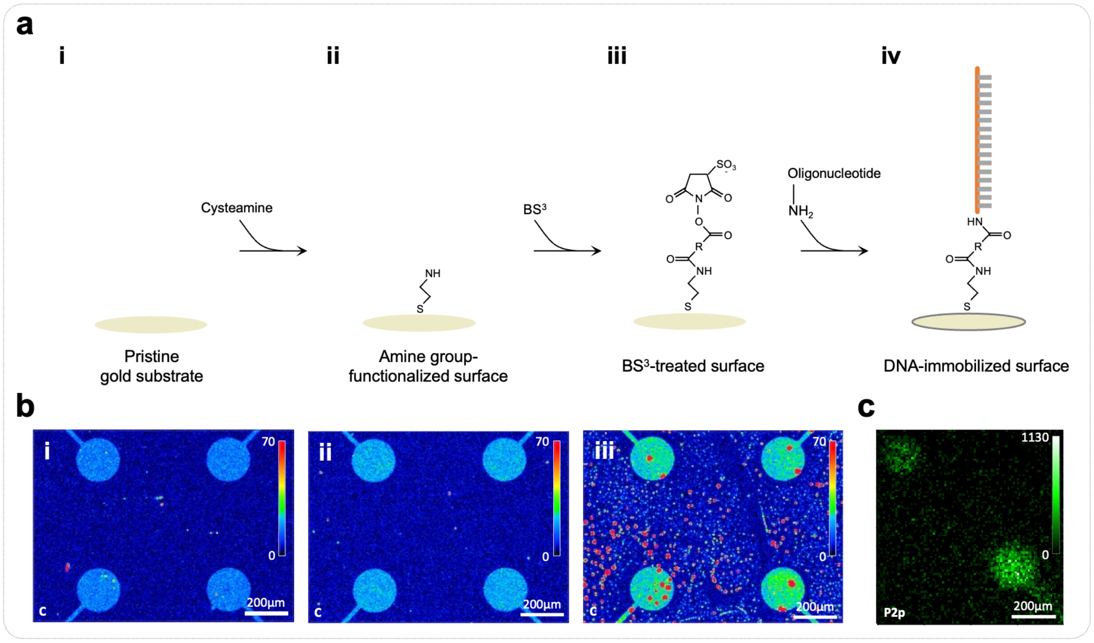

Supplementary Fig. 1 Surface functionalization and selective electric field-guided DNA immobilization. (a) Schematic illustration of the surface functionalization process on a gold microarray, (i. Pristine gold microarray, ii. Amine group-functionalized gold microarray via cysteamine, iii. Bis(sulfosuccinimidyl)suberate (BS^3)-treated surface, and iv. Electric field-guided DNA immobilization) **(b)** Electron probe microanalyzer (EPMA) mapping images of carbon (C) at each functionalization step (i-iii), **(c)** Phosphorus signal (P2p) X-ray photoelectron spectroscopy (XPS) mapping image of selective electric field-guided DNA immobilization in a checkerboard configuration.

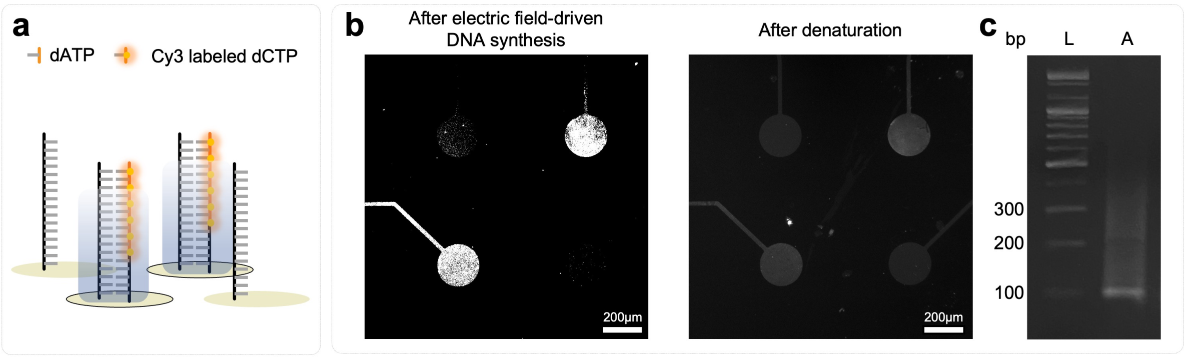

Supplementary Fig. 2 Fluorescence-based monitoring of electric field-driven DNA synthesis. (a) Schematic of electric field-driven DNA synthesis on poly(TG) template using Cy3-labeled dCTP and dATP, **(b)** Fluorescence microscopy images of the 2×2 electrode array following electric field-guided synthesis (left) and denaturation (right), demonstrating spatially selective and efficient DNA synthesis through distinct fluorescent signals in a checkerboard pattern under electric field conditions (scale bars, 200 μm), and **(c)** A gel electrophoresis result of the synthesized DNA (L, DNA ladder; A, amplified sample)

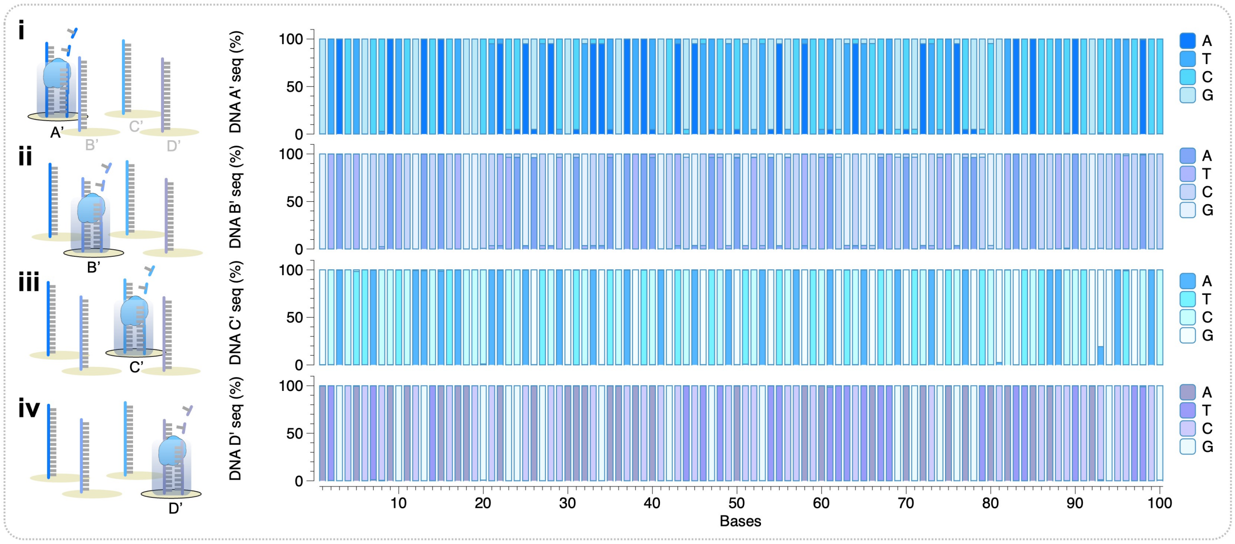

Supplementary Fig. 3 NGS consensus results for random access to four distinct DNA sequences(i-iv), corresponding to A', B', C', and D', respectively.

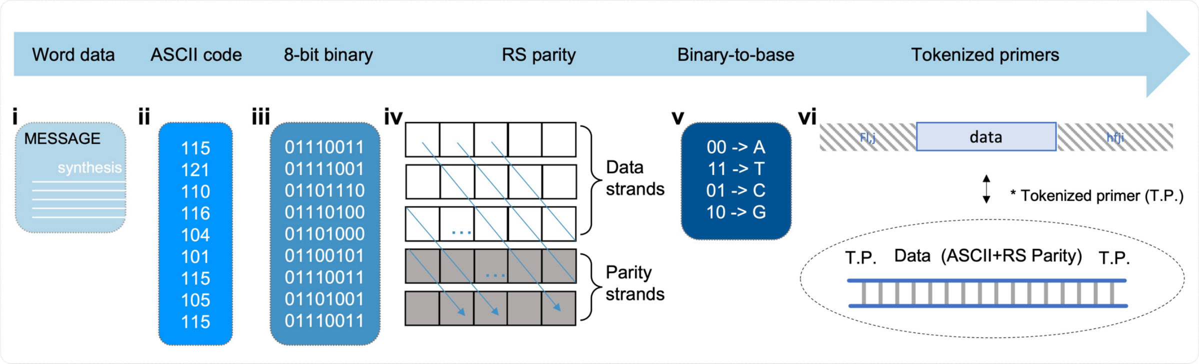

Supplementary Fig. 4. Word-based DNA encoding process. (i) Word data, **(ii)** ASCII code conversion, **(iii)** 8-bit binary transformation, **(iv)** Reed-Solomon (RS) parity generation, **(v)** binary-to-base translation, and **(vi)** tokenized primer (T.P.) addition.

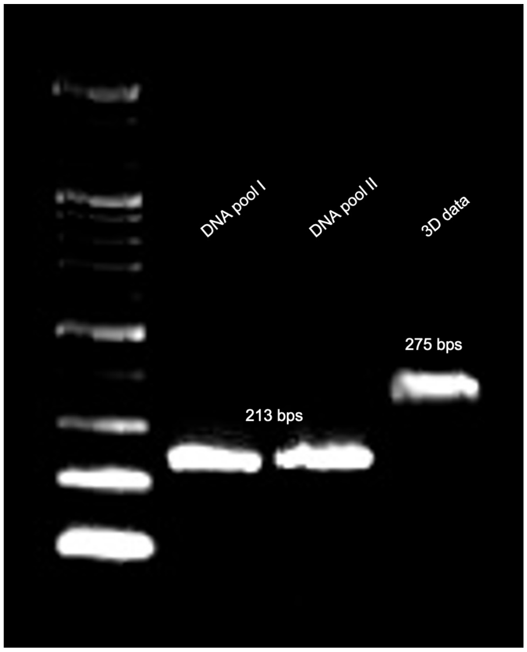

Supplementary Fig. 5. Gel electrophoresis analysis of the ligated DNA pools.

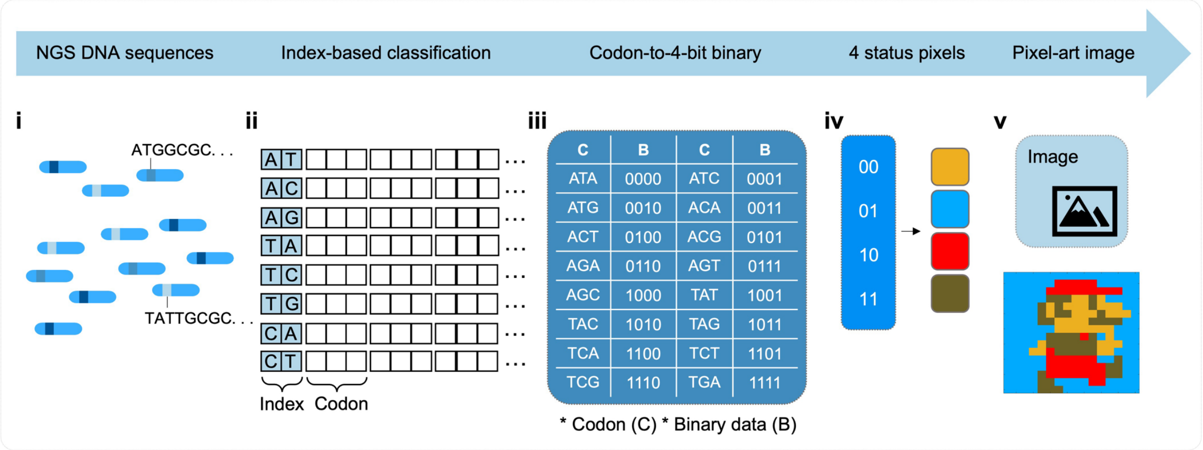

Supplementary Fig. 6. Pixel-based DNA data decoding process. (i) NGS DNA sequences, **(ii)** primer-based sorting and index classification, **(iii)** codon-to-4-bit binary translation, **(iv)** two-pixel mapping for four color states (00, 01, 10, 11), and **(v)** pixel-art image reconstruction.

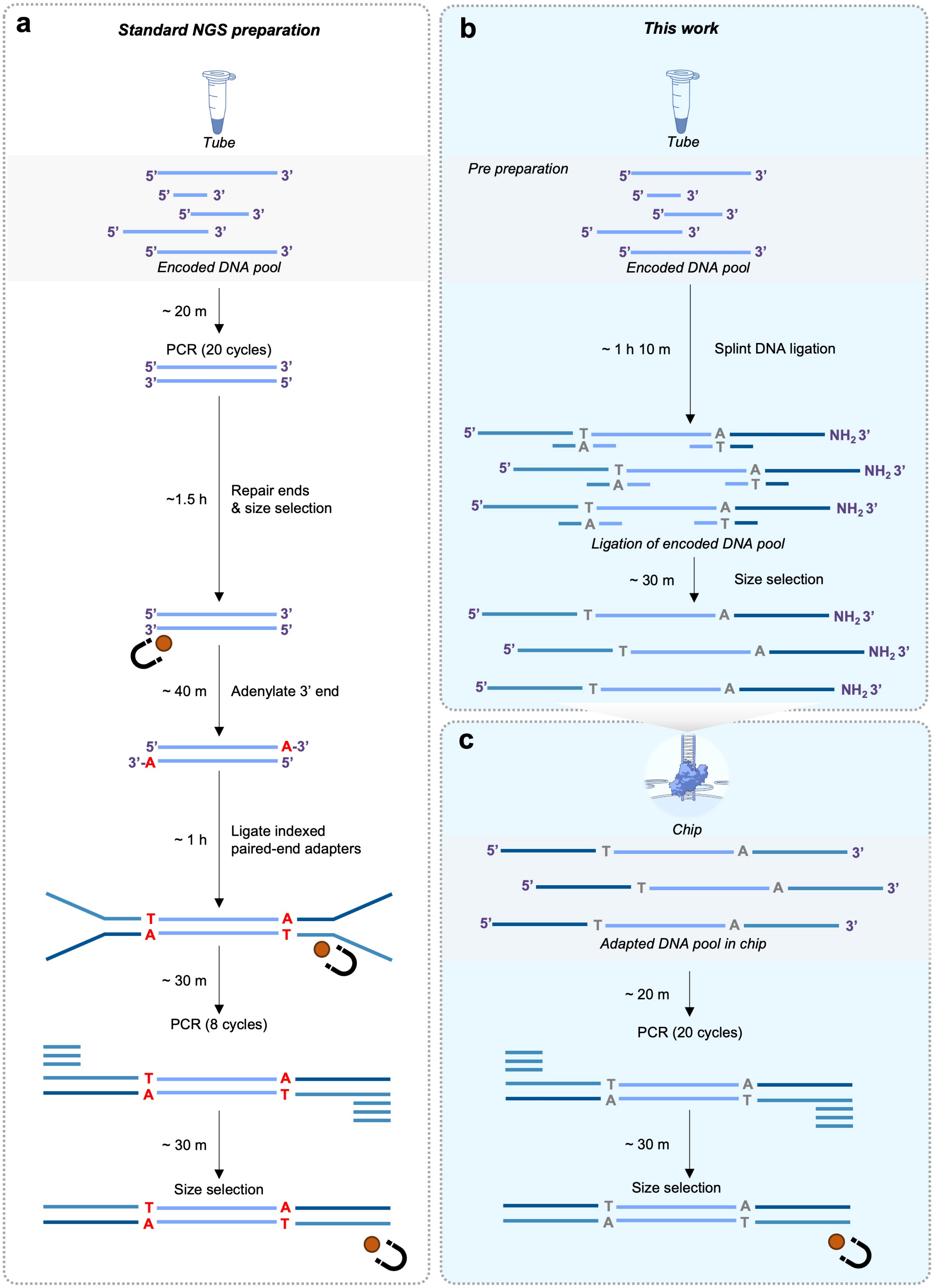

Supplementary Fig. 7 Protocol for NGS library preparation from adapted DNA pool in chip compared with standard NGS sample preparation. (a) Schematic of the protocol timeline for conventional NGS library preparation, and **(b, c)** Protocol timeline for NGS library preparation from adapted DNA pool in chip.

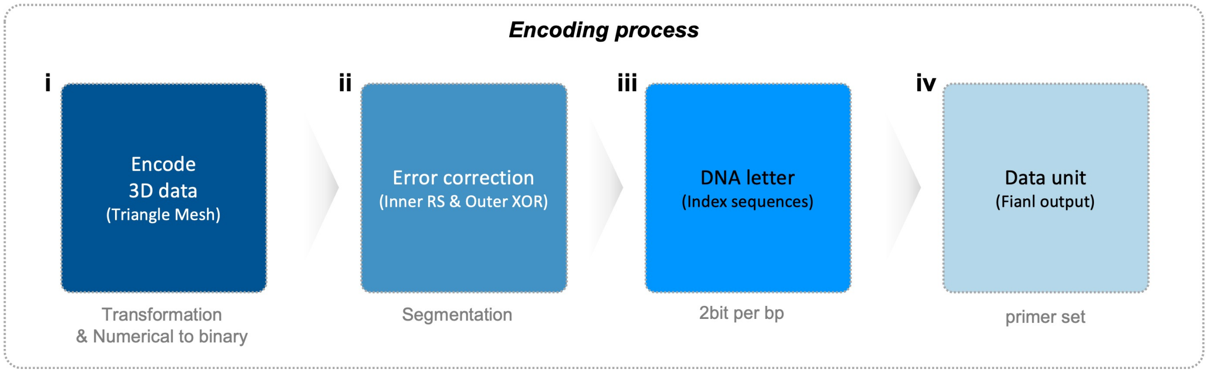

Supplementary Fig. 8 Simple schematic representation of the 3D data encoding workflow used for our DNA storage. (i) Transformation of 3D structural information, including Cartesian coordinates and triangular mesh vectors, into numerical and binary formats, **(ii)** Application of RS and XOR-based error correction codes to improve data reliability, **(iii)** Conversion into DNA letter sequences (2 bits per base), and **(iv)** Segmentation into 150-base DNA packets containing 20-base primer sets and index sequences.

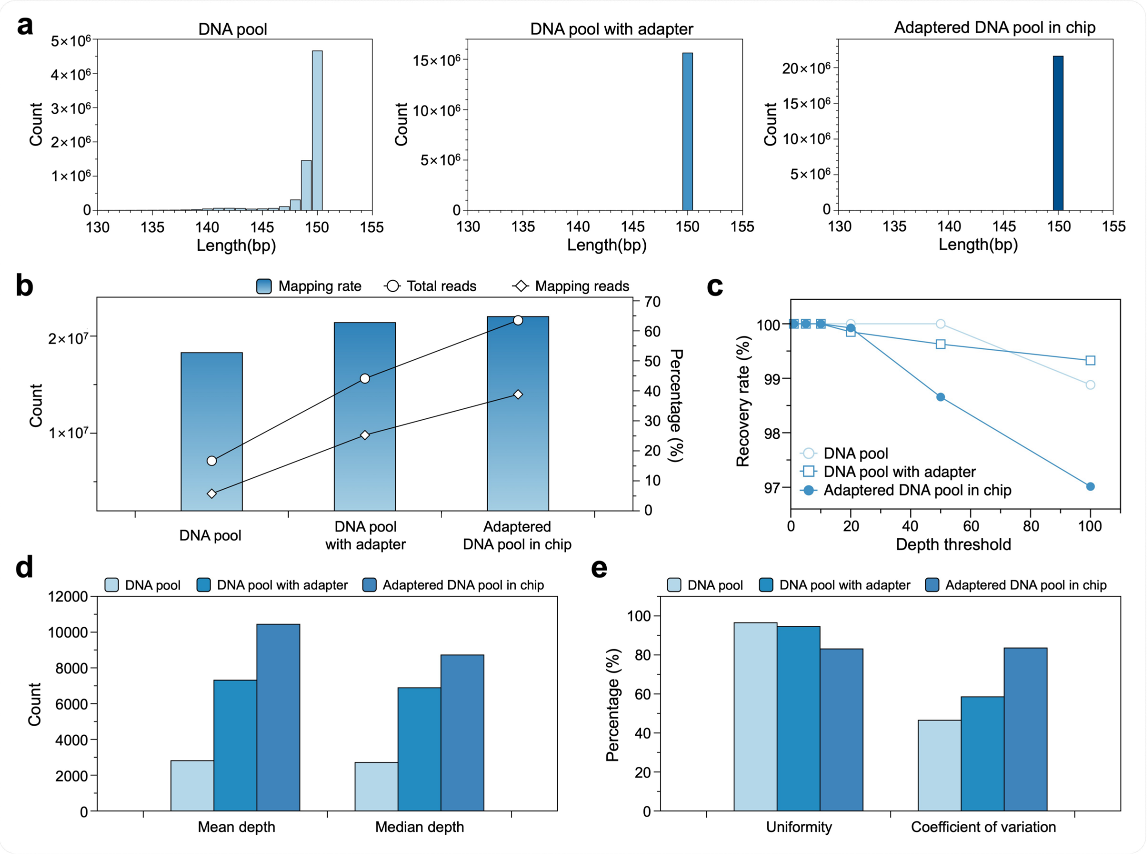

Supplementary Fig. 9 Comparative analysis of NGS results from three DNA pool samples. **(a) – (e)** NGS analysis comparing the DNA pool, DNA pool with adapter, and adaptered DNA pool in chip. These analyses show **(a)** the length distribution of sequencing reads, **(b)** the total reads, mapped reads, and mapping rates, **(c)** the recovery rate as a function of depth threshold, **(d)** the mean and median sequencing depths, and **(e)** uniformity and the coefficient of variation (CV) of sequencing coverage.

Table S1. Comparison of DNA data storage platforms for random access.

Parameter	This work	Kang et al. (*35*)	Jiang et al. (*36*)	Banal et al. (19)	Bögels et al. (*20*)	Mao et al. (*21*)	Antkowiak et al. (*23*)	Liu et al. (*22*)
**Platform**	Microelectrode array	Glass substrate (SiO_2)	Nylon-polyester cassette tape and ZIFs	Silica capsule	BSA-PNIPAm proteinosome	Microfluidics and SiO_2 NPs and ZIF-8 MOF	Silica capsule	Photonic microsphere
**Data access**	Electric field-addressable electrodes	2D molecular QR code	Linear tape file system with 1D barcode	FACS	PCR/ FACS	FACS	EWOD	Microscopy-spectrometer (sterile surgical scalpel)
**Molecule density (copies/nm²)**	0.035	-	4.54×10^{-4}	N/A*	N/A*	8×10^{-5}	-	0.135[a]
**Data orthogonality by primer**	Yes	No	Yes	No	No	No	No	No
**Pool selection method**	Yes (electric field-driven elongation)	No (elongation)	No (elongation)	Yes (fluorescent tagged sequence)	Yes (primer/ fluorescent tagged sequence)	Yes (barcoded MOF)	No (electrowetting)	Yes (photonic MOF)
**NGS library in platform**	Yes	No	No	No	No	No	No	No
**Crosstalk**	Very low (0.11%†)	Low	Very low (0.03%)	Low	Low	Low (4%)	-	Very low (0.0005%)
**Immobilization and capsulation time**	20 min	2 h	21 min	2 h	~6 day	40 min	~6 day	~1 h 20 min
**Random access and DNA extraction time**	2 min	50 min	6 min	50 min	1 h 15 min	5 min	5 min	45 min
**NGS library preparation time**	50 min‡	6 h	2 h	6 h	6 h	2 h	6 h	6 h
**Reusability**	100 cycles (10^5 projected)	-	10 cycles	-	4 cycles	10 cycles (84 projected)	-	-

* 3D architecture; 2D surface density not measurable.

† 0.11% with different primers; 6% with identical primers across electrodes.

‡ Direct NGS compatibility without standard library preparation protocol.

[a] The BET specific surface area is 7.010 m^2/g, calculated based on assumed parameters.

Abbreviations: FACS, fluorescence-activated sorting; MOF, metal-organic framework; EWOD, electrowetting-on-dielectric; BSA, bovine serum albumin; PNIPAm, poly(N-isopropylacrylamide); ZIFs, zeolitic imidazolate frameworks

Table S2. Comparison of DNA data storage platforms for writing with this work.

Feature	Nguyen et al. (*27*)	Xu et al. (*28*)	Li et al. (*25*)	Newman et al. (*26*)	This work
**DNA data storage**	Single sequence synthesis (W), NGS (R)	Single sequence synthesis (W), electrochemical sensing (R)	Single sequence synthesis (W), storage (S), pyrosequencing, NGS (R)	Multiple sequence storage (W), retrieval (RA), NGS (R)	Multiple sequence encoding (W), retrieval (RA), NGS (R)
**Architecture**	Nanoelectrode wells (diameter = 650 nm, depth = 200 nm)	Au NPs-modified gold electrodes (diameter = 260 or 500 μm), SlipChip	Magnetic beads (diameter = 0.6 μm) DMF electrodes ($2.41\times2.41\ mm^2$)	DMF electrodes ($2\times2.7\ mm^2$)	Microelectrode array gold electrodes (diameter = 200 μm)
**Writing method**	Chemistry (phosphoramidite)	Chemistry (phosphoramidite)	Enzyme (E-ZaTdT)	Resuspension	Electrical field-driven elongation
**Sequence per electrode**	1 sequence	1 sequence	1 sequence	2024 sequences	1,339 sequences
**Max oligo length**	104 nt	43 nt	18 nt	315 nt	275 nt
**Data capacity per electrode**	0.040 KB	0.020 KB	0.028 KB	20 KB	198.8 KB
**Data density per dimension**	$0.120\ KB/\mu m^2$	$1.019\times10^{-7}\ KB/\mu m^2$	$4.8208\times10^{-9}\ KB/\mu m^2$	$3.703\times10^{-6}\ KB/\mu m^2$	$0.006\ KB/\mu m^2$
**Reusability**	No	No	No	No	Yes
**Electrical control principle**	Electrochemical deprotection	Electrochemical deprotection	Electrowetting	Electrowetting	Electric field hybridization and production
**Parallel capability**	4-plex	4-plex	96-plex	127-plex	16-plex

(W): Writing
(S): storage
(RA): Random access
(R): Reading

Abbreviations: Au NPs, gold nanoparticles; DMF, digital microfluidics; E-ZaTdT, engineered terminal deoxynucleotidyl transferase; KB, kilobytes; NGS, next-generation sequencing; nt, nucleotides;

REFERENCES

1. Y. Hao, Q. Li, C. Fan, F. Wang, Data storage based on DNA. *Small Struct.* **2**, 2000046 (2021).

2. R. N. Grass, R. Heckel, M. Puddu, D. Paunescu, W. J. Stark, Robust chemical preservation of digital information on DNA in silica with error-correcting codes. *Angew. Chem. Int. Ed. Engl.* **54**, 2552–2555 (2015).

3. S. Wang, X. Mao, F. Wang, X. Zuo, C. Fan, Data storage using DNA. *Adv. Mater.* **36**, e2307499 (2024).

4. G. M. Church, Y. Gao, S. Kosuri, Next-generation digital information storage in DNA. *Science* **337**, 1628–1628 (2012).

5. L. Organick, S. D. Ang, Y.-J. Chen, R. Lopez, S. Yekhanin, K. Makarychev, M. Z. Racz, G. Kamath, P. Gopalan, B. Nguyen, C. N. Takahashi, S. Newman, H.-Y. Parker, C. Rashtchian, K. Stewart, G. Gupta, R. Carlson, J. Mulligan, D. Carmean, G. Seelig, L. Ceze, K. Strauss, Random access in large-scale DNA data storage. *Nat. Biotechnol.* **36**, 242–248 (2018).

6. S. Yang, B. W. A. Bögels, F. Wang, C. Xu, H. Dou, S. Mann, C. Fan, T. F. A. de Greef, DNA as a universal chemical substrate for computing and data storage. *Nat. Rev. Chem.* **8**, 179–194 (2024).

7. K. Li, H. Chen, D. Li, C. Yang, H. Zhang, Z. Zhu, Empowering DNA-based information processing: Computation and data storage. *ACS Appl. Mater. Interfaces* **16**, 68749–68771 (2024).

8. B. Wang, S. S. Wang, C. Chalk, A. D. Ellington, D. Soloveichik, Parallel molecular computation on digital data stored in DNA. *Proc. Natl. Acad. Sci. U.S.A.* **120**, e2217330120 (2023).

9. C. Bee, Y.-J. Chen, M. Queen, D. Ward, X. Liu, L. Organick, G. Seelig, K. Strauss, L. Ceze, Molecular-level similarity search brings computing to DNA data storage. *Nat. Commun.* **12**, 4764 (2021).

10. J. Zhang, C. Hou, C. Liu, CRISPR-powered quantitative keyword search engine in DNA data storage. *Nat. Commun.* **15**, 2376 (2024).

11. D. Reinsel, J. Gantz, J. Rydning, “White paper on The digitization of the world from edge to core (IDC, 2018).

12. D. Bar-Lev, O. Sabary, E. Yaakobi, The zettabyte era is in our DNA. *Nat. Comput. Sci.* **4**, 813–817 (2024).

13. P. Y. De Silva, G. U. Ganegoda, New trends of digital data storage in DNA. *Biomed. Res. Int.* **2016**, 8072463 (2016).

14. Y. Zhou, K. Bi, Q. Ge, Z. Lu, Advances and challenges in random access techniques for in vitro DNA data storage. *ACS Appl. Mater. Interfaces* **16**, 43102–43113 (2024).

15. C. Winston, L. Organick, D. Ward, L. Ceze, K. Strauss, Y.-J. Chen, Combinatorial PCR method for efficient, selective oligo retrieval from complex oligo pools. *ACS Synth. Biol.* **11**, 1727–1734 (2022).

16. A. El-Shaikh, M. Welzel, D. Heider, B. Seeger, High-scale random access on DNA storage systems. *NAR Genom. Bioinform.* **4**, lqab126 (2022).

17. C. N. Takahashi, B. H. Nguyen, K. Strauss, L. Ceze, Demonstration of end-to-end automation of DNA data storage. *Sci. Rep.* **9**, 4998 (2019).

18. M. H. Raza, S. Desai, S. Aravamudhan, R. Zadegan, An outlook on the current challenges and opportunities in DNA data storage. *Biotechnol. Adv.* **66**, 108155 (2023).

19. J. L. Banal, T. R. Shepherd, J. Berleant, H. Huang, M. Reyes, C. M. Ackerman, P. C. Blainey, M. Bathe, Random access DNA memory using Boolean search in an archival file storage system. *Nat. Mater.* **20**, 1272–1280 (2021).

20. B. W. A. Bögels, B. H. Nguyen, D. Ward, L. Gascoigne, D. P. Schrijver, A.-M. Makri Pistikou, A. Joesaar, S. Yang, I. K. Voets, W. J. M. Mulder, A. Phillips, S. Mann, G. Seelig, K. Strauss,

Y.-J. Chen, T. F. A. de Greef, DNA storage in thermoresponsive microcapsules for repeated random multiplexed data access. *Nat. Nanotechnol.* **18**, 912–921 (2023).

21. C. Mao, S. Wang, J. Li, Z. Feng, T. Zhang, R. Wang, C. Fan, X. Jiang, Metal–organic frameworks in microfluidics enable fast encapsulation/extraction of DNA for automated and integrated data storage. *ACS Nano* **17**, 2840–2850 (2023).

22. Q.-J. Liu, Q. Liu, J. Zhang, C. Zhang, L. Qian, H. Qi, Y.-S. Li, D.-P. Song, Photonic microspheres for high-capacity DNA data storage: Robust, straightforward, and scalable random access via nonfading indexes. *Sci. Adv.* **11**, eadw2613 (2025).

23. P. L. Antkowiak, J. Koch, B. H. Nguyen, W. J. Stark, K. Strauss, L. Ceze, R. N. Grass, Integrating DNA encapsulates and digital microfluidics for automated data storage in DNA. *Small* **18**, e2107381 (2022).

24. J. A. Smith, B. H. Nguyen, R. Carlson, J. G. Bertram, S. Palluk, D. H. Arlow, K. Strauss, Spatially selective electrochemical cleavage of a polymerase-nucleotide conjugate. *ACS Synth. Biol.* **12**, 1716–1726 (2023).

25. K. Li, X. Lu, J. Liao, H. Chen, W. Lin, Y. Zhao, D. Tang, C. Li, Z. Tian, Z. Zhu, H. Jiang, J. Sun, H. Zhang, C. Yang, DNA-DISK: Automated end-to-end data storage via enzymatic single-nucleotide DNA synthesis and sequencing on digital microfluidics. *Proc. Natl. Acad. Sci.* **121**, e2410164121 (2024).

26. S. Newman, A. P. Stephenson, M. Willsey, B. H. Nguyen, C. N. Takahashi, K. Strauss, L. Ceze, High density DNA data storage library via dehydration with digital microfluidic retrieval. *Nat. Commun.* **10**, 1706 (2019).

27. B. H. Nguyen, C. N. Takahashi, G. Gupta, J. A. Smith, R. Rouse, P. Berndt, S. Yekhanin, D. P. Ward, S. D. Ang, P. Garvan, H.-Y. Parker, R. Carlson, D. Carmean, L. Ceze, K. Strauss, Scaling DNA data storage with nanoscale electrode wells. *Sci. Adv.* **7**, eabi6714 (2021).

28. C. Xu, B. Ma, Z. Gao, X. Dong, C. Zhao, H. Liu, Electrochemical DNA synthesis and sequencing on a single electrode with scalability for integrated data storage. *Sci. Adv.* **7**, eabk0100 (2021).

29. D. Lim, S. Noh, T. Kang, N. Nergui, H. S. Eun, Y. Song, Amine-to-amine deoxyribonucleic acid conjugation process on gold surfaces for electric field-assisted hybridization. *Langmuir* **41**, 7008–7015 (2025).

30. Y. Song, S. Kim, M. J. Heller, X. Huang, DNA multi-bit non-volatile memory and bit-shifting operations using addressable electrode arrays and electric field-induced hybridization. *Nat. Commun.* **9**, 281 (2018).

31. Y. Ma, Z. Zhang, B. Jia, Y. Yuan, Automated high-throughput DNA synthesis and assembly. *Heliyon* **10**, e26967 (2024).

32. I. Y. Wong, N. A. Melosh, Directed hybridization and melting of DNA linkers using counterion-screened electric fields. *Nano Lett.* **9**, 3521–3526 (2009).

33. C. Zhang, R. Wu, F. Sun, Y. Lin, Y. Liang, J. Teng, N. Liu, Q. Ouyang, L. Qian, H. Yan, Parallel molecular data storage by printing epigenetic bits on DNA. *Nature* **634**, 824–832 (2024).

34. M. Yu, D. Lim, J. Kim, Y. Song, Processing DNA storage through programmable assembly in a droplet-based fluidics system. *Adv. Sci.* **10**, e2303197 (2023).

35. T. Kang, D. Lim, W. Lee, Y. Song, Polymerase elongation onto patterned DNA for random accessed DNA data storage. *Biochip J.* **19**, 636–648 (2025).

36. J. Li, C. Mao, S. Wang, X. Li, X. Luo, D. Wang, S. Zheng, J. Shao, R. Wang, C. Fan, X. Jiang, A compact cassette tape for DNA-based data storage. *Sci. Adv.* **11**, eady3406 (2025).
